# Aerosol-induced brucellosis increases TLR-2 expression and increased complexity in the microanatomy of astroglia in rhesus macaques

**DOI:** 10.3389/fcimb.2013.00086

**Published:** 2013-12-02

**Authors:** Kim M. Lee, Kevin B. Chiu, Hope A. Sansing, Peter J. Didier, Thomas A. Ficht, Angela M. Arenas-Gamboa, Chad J. Roy, Andrew G. MacLean

**Affiliations:** ^1^Program in Biomedical Science, Tulane School of MedicineNew Orleans, LA, USA; ^2^Divisions of Comparative Pathology and Microbiology, Tulane National Primate Research CenterCovington, LA, USA; ^3^Department of Biomedical Science, Tulane UniversityNew Orleans, LA, USA; ^4^Veterinary Pathobiology, Texas A&M UniversityCollege Station, TX, USA; ^5^Department of Microbiology & Immunology, Tulane School of MedicineNew Orleans, LA, USA

**Keywords:** Brucella, gliosis, innate immune activation, toll-like receptor, astrocyte

## Abstract

*Brucella melitensis*, a bacterial pathogen and agent of epizootic abortion causes multiple pathologies in humans as well as a number of agriculturally important animal species. Clinical human brucellosis manifests as a non-specific, chronic debilitating disease characterized by undulant fever, arthropathies, cardiomyopathies and neurological *sequelae*. These symptoms can occur acutely for a few weeks or persist for months to years. Within the brain, endothelial and glial cells can be infected leading to downstream activation events including matrix metalloprotease (MMP) and cytokine secretion and Toll-like receptor (TLR) signaling. These events are likely to lead to tissue remodeling, including morphologic changes in neuronal and glial cells, which are linked to neurological complications including depressive behavior, immune activation and memory loss. Our hypothesis was that *B. melitensis* infection and neurobrucellosis would lead to activation of astrocytes through upregulation of TLR2 and stimulate concurrent changes in the microanatomy. All six animals were infected via inhalation route. TLR2 expression was approximately doubled in white matter astrocytes of infected rhesus macaques. There was also a 50% increase in the number of astrocytes per unit area in subcortical white matter tracts suggesting increased innate immune activation. This coincided with dramatic increases in the length and complexity of the cell arbor of hypertrophic astrocytes in both cortical gray and white matter. Thus, aerosol-induced brucellosis results in dramatically increased innate immune activation of astrocytes in the absence of widespread neuroinflammation.

## Introduction

Brucellosis is a zoonotic disease, caused by the bacterial pathogen *Brucella* spp. There are ten species based on the preferential host specificity. Of the known species, most have been shown to infect humans, but due to close association with certain animals human infection is generally restricted to *Brucella melitensis* (goats and sheep), *B. abortus* (cattle), *B. suis* (swine), and *B. canis* (dogs) (Boschiroli et al., 2001). Brucellosis is acquired by consumption of unpasteurized dairy products or by contact with infected animals. The disease is harbored mainly in domesticated animal reservoirs, although transmission through wild animals has also been known to occur, and persistence in the environment remains a concern (Pappas, 2010). While rare, human-to-human transmission has been documented through close contact, such as sexual transmission and from mother to child transmission (Ceylan et al., 2012). The most virulent species in human infection is *B. melitensis*, followed by *Brucella suis*, *B. abortus*, and *B. canis* (Pappas et al., 2005; Pei et al., 2012). *B. melitensis* has been shown to be infectious by multiple routes, including passive inhalation of small particle aerosol at relatively low doses. The potential for misuse of *B. melitensis* is a concern, since infection via aerosol requires a relatively low dose of bacteria (10–50 bacteria), and the process of producing *Brucella* is not technically difficult (Yagupsky and Baron, 2005). As such, it is currently listed as a category B priority pathogen by the U.S. National Institutes of Health, and has also been designated as a select agent by the U.S. Centers for Disease Control and Prevention and the U.S. Department of Agriculture. Due to its ability to cause disease, low infectivity dose, zoonotic potential and the fact that a model for aerosolized *B. melitensis* infection in rhesus macaque has already been established (Henning et al., 2012), *B. melitensis* was chosen for this study.

In humans, the most common indication of a *Brucella* infection is an undulating fever, also known as Malta fever, where febrile symptoms exhibit every few days (Corbel, 2006). Other symptoms of infection are Protean and non-specific and include but are not limited to malaise, fatigue, headache, stomach problems, weight loss, and achy joints, thus making brucellosis difficult to diagnose. While most symptoms are not life-threatening, chronic infection can result from untreated or unsuccessfully treated infections, where chronic fatigue syndrome symptoms can continue for years. Furthermore, complications from persistent infection can affect much of the body, resulting in gastrointestinal, respiratory, cardiac, and neurological symptoms (Corbel, 2006).

Endothelial cells can be productively infected by *Brucella* species with concomitant upregulation of integrins and secretion of IL-6 (Ferrero et al., 2011). Similarly, astrocytes can be activated by either heat-killed bacteria or lipoproteins to secrete chemokines, proliferate or enter apoptosis (Baldi and Giambartolomei, 2012). Recently, astrocyte infection by *Brucella* has been shown to induce matrix metalloproteases (MMP) in a TNF-a dependent manner (Miraglia et al., 2013). The MMPs are known to induce tissue remodeling (Bixby and Harris, 1991; Abdul Muneer et al., 2012), including in the brain (Szklarczyk et al., 2002). Extensive changes in the microanatomy of astrocytes in frontal cortex have been reported in suicidal humans (Torres-Platas et al., 2011) and macaques with self-injurious behavior (Lee et al., 2013) or infected with Simian Immunodeficiency Virus (Lee et al., under revision). These changes coincided with increased expression of TLR2 protein, and thus innate immune activation of astrocytes.

Due to the chronic nature of *Brucella* infection, it is likely that there will either be long term activation of astrocytes or multiple rounds of activation. We have recently shown that the sequence of activation is important in the activation profiles of astrocytes (Renner et al., 2013). Neurological *sequelae* to *Brucella* infection are common, with changes in cortex reported in humans, characterized by multiple discrete and confluent white matter lesions visible by MRI involving the periventricular and the subcortical white matter (Nalini et al., 2012). What is not known, however, is whether there are neurological complications associated with early infection events.

Our hypothesis was that *B. melitensis* infection would lead to activation of astrocytes through upregulation of TLR2 and concurrent changes in the microanatomy. In this study, we measured astrocyte activation in response to acute *B. melitensis* infection. TLR2 expression approximately doubled in white mater astrocytes of India-origin rhesus macaques infected with *B. melitensis*. This coincided with astrocyte hypertrophy with increased process length and complexity in a manner distinct from other diseases previously examined.

## Materials and methods

### Ethics statement, animal housing and selection of tissues

Animals were maintained in Animal Biosafety Level 3(BSL-3) housing with a 12:12-h light:dark cycle, relative humidity 30–70%, and a temperature of 17.8–28.9°C. Water was available *ad libitum*, and a standard commercially formulated nonhuman primate diet (Lab Fiber Plus Monkey DT, 5K63, PMI Nutrition International, St. Louis, MO) was provided twice daily and supplemented daily with fresh fruit and/or forage material as part of the environmental enrichment program. All animals at TNPRC have environmental enrichment, widely used to improve welfare in captive macaques. Over the course of their life times, all subjects experienced some pair or group housing as well as periods of single housing. Each cage (Allentown, Inc., Allentown, NJ) measured 36 inches (91.4 cm) in height with 8.6 square feet (0.8 square meters) of floor space and contained a perch, a portable enrichment toy, a mirror, and a forage board for feeding enrichment. Practices in the housing and care of animals conformed to the regulations and standards of the PHS Policy on Humane Care and Use of Laboratory Animals, and the Guide for the Care and Use of Laboratory Animals. The Tulane National Primate Research Center (Animal Welfare Assurance # A4499-01) is fully accredited by the Association for the Assessment and Accreditation of Laboratory Animal Care-International. All animals are routinely cared for according to the guidelines prescribed by the NIH Guide to Laboratory Animal Care. The TNPRC conducts all research in accordance with the recommendations of the Weatherall report -“The use of non-human primates in research.” The Institutional Animal Care and Use Committee (IACUC) of the Tulane National Primate Research Center approved all animal-related protocols, including any treatments used with nonhuman primates (protocol number p-0129). All animal procedures were overseen by veterinarians and their staff.

### Animals, tissues and infection with *Brucella*

Tissue from five uninfected control and six infected Indian-origin rhesus macaques (*Macaca mulatta*) were used for this study, for a total of 11 animals. All of the animals were infected with *B. melitensis* by inhalation with between 8.5 × 10^5^ and 1.3 × 10^6^ cfu, as specified in Table 1. Aerosol infection of animals was performed singly using a fully automated inhalation exposure system (Biaera Technologies, Hagerstown, MD) that utilizes a dynamic (flow through) 16 liter head-only exposure chamber and aerosol generator (collison nebulizer, BGI, Inc., Waltham, MA) that produces highly respirable (≈2 μm) bioaerosols. The aerosol exposures are acute (10 min) and is performed with anesthetized animals in dorsal recumbency. The inhalation apparatus is enclosed within a Class III biological safety cabinet housed within a BSL-3 laboratory environment; animals are transported into and out of the Class III BSC singly for exposure to the bacterial agent. This aerosol infection with *Brucella* is in agreement with studies by other groups (Henning et al., 2012), and with other infectious agents at TNPRC (Hartings and Roy, 2004; Mehra et al., 2011; Verreault et al., 2012, 2013; Roy et al., 2013). All animals were humanely euthanized 45 days postinfection with an intravenous overdose of pentobarbital and tissues collected at necropsy.

**Table 1:** **Bacterial loads in blood and selected tissues in macaques exposed to *B. melitensis***.

**Dose group (*n*) (Aerosol dose)**	**Day pi**		**Blood (CFU/ml)**	**BAL (CFU/ml)**	**Liver (CFU/mg tissue)**	**Bronch. LN (CFU/mg tissue)**
A (3) (1.0 × 10^5^)^a^ 8.5 × 10^5^± 2.1 × 10^5^ (mean ± std.)	+1	A	8.3 × 10^2^ ± 5.0 × 10^2^ (3/3)^b^	ND	ND	ND
		B	1.5 × 10^3^ ± 1.3 × 10^3^ (3/3)	ND	ND	ND
B (3) (1.0 × 10^6^)^a^ 1.3 × 10^6^ ± 3.4 × 10^5^ (mean ± std.)	+15	A	LOD	1.0 × 10^2^ ± 8.1 × 10^1^ (3/3)	ND	2.8 × 10^4^ ± 3.0 × 10^4^ (2/3)
		B	LOD	9.8 × 10^2^ ± 8.6 × 10^2^ (3/3)	5.2 × 10^3^ ± 3.2 × 10^3^ (3/3)	LOD
	+45	A	LOD	1.2 × 10^3^ ± 1.3 × 10^3^ (2/3)	2.8 × 10^4^ ± 7.5 × 10^3^ (3/3)	7.9 × 10^4^ ± 3.3 × 10^4^ (3/3)
		B	LOD	1.7 × 10^4^ ± 1.1 × 10^4^ (2/3)	2.5 × 10^4^ ± 3.5 × 10^4^ (3/3)	2.4 × 10^4^ ± 1.5 × 10^4^ (3/3)

a CFU/animal (target aerosol dose).

b (number of positive animals/group).

LOD under the limit of detection.

N.D. not done.

All animals were housed at the Tulane National Primate Research Center in accordance with the standards of the Association for Assessment and Accreditation of Laboratory Animal Care and the “Guide for the Care and Use of Laboratory Animals” prepared by the National Research Council, National Academic Press, Washington, DC. The Tulane Institutional Animal Care and Use Committee approved all studies. All animals were humanely euthanized at the predetermined terminal endpoint of the study and tissues collected at necropsy.

### Histologic examination of tissues

Formalin-fixed, paraffin-embedded tissues were sectioned at 6 μm and mounted onto positively charged glass slides. Sections were baked for 1 h at 60°C, deparaffinized in xylene, and then rehydrated in graded concentrations of ethanol. Slides were stained with hematoxylin and eosin to allow histologic examination of the tissues. Collected brain sections included brainstem, cerebellum and cerebrum. Other tissues examined included, adrenal, bone marrow, colon, duodenum, esophagus, eyes, gall bladder, heart, ileocecal junction, jejunum, kidneys, large intestine, liver, lung, lymph nodes (axillary, bronchial, inguinal, mesenteric, submandibular), pancreas, salivary gland, seminal vesicle, skeletal muscle, eyelid, small intestine, spinal cord, spleen, stomach, testes, thymus, thyroid, tongue, tonsil, and trachea.

### Immunohistochemistry

Based on our previous studies of animals infected with viruses (SIV and Dengue) or with abnormal behavior models (Lee et al., 2013), we were interested to determine if there was increased innate immune activation on astrocytes of animals infected with *B. melitensis*. Formalin-fixed, paraffin-embedded tissues of frontal cortex of all eleven animals were sectioned at 6 μm and mounted onto positively charged glass slides. Sections were baked for 1 h at 60°C, deparaffinized in xylene, and then rehydrated in graded concentrations of ethanol. Antigen retrieval was carried out for 20 min using a microwave on high power and a citrate-based antigen unmasking solution (Vector Labs, Burlingame, CA). Tissues were blocked in a 10% (v/v) normal goat serum solution (Invitrogen, Carlsbad, CA) for 1 h at room temperature before antibodies were applied. Tissues were incubated with GFAP primary antibody (GA-5, Sigma) and TLR2 (Abcam) overnight at 4°C, washed three times with PBS with 2% (w/v) bovine serum albumin (Santa Cruz, CA, PBS/BSA), and then incubated in the dark for 60 min at room temperature with secondary antibodies directly conjugated with Alexa 488 (green) or Alexa 568 (red) (Molecular Probes/Invitrogen, Carlsbad, CA). Sections were washed three times in PBS/FSG, cover-slipped (CrystalCruz, Santa Cruz) with Prolong Gold with DAPI (Molecular Probes/Invitrogen), and imaged on a Nikon Eclipse TE2000-U microscope.

### Quantification of TLR2 expression

Images of double-labeled (GFAP and TLR2) sections in non-overlapping fields were captured by fluorescence microscopy (Nikon Eclipse TE2000-U). An average of five fields of astrocytes were imaged separately for both white and gray matter at 20×, and the number of GFAP and TLR2 double-labeled astrocytes was quantified and expressed as a percentage of the total number of GFAP-labeled cells. Statistics were performed using GraphPad Prism (version 5, GraphPad Software). Comparisons were made by One-Way ANOVA with Tukey's *post-hoc* multiple comparison test to determine significance between groups. Results are expressed as mean ± SEM. Significance was set at *p* < 0.05.

### Quantification of astrocyte morphology

**Figure 1:**
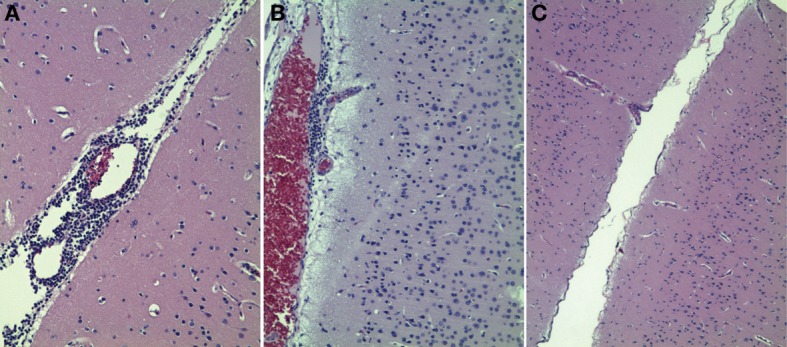
**Histologic examination of macaque brain tissues of animals infected with *B. melitensis.*** Two of six animals infected with *Brucella* had evidence of focally mild to moderate nonsupperative encephalitis **(A,B)**. Four of six animals had no histologic evidence of encephalitis **(C)**.

All samples were coded and analyzed randomly by a researcher blinded to animal number and condition. Images of non-overlapping fields in frontal and/or parietal cortical sections were captured by fluorescence microscopy at 40× objective (Nikon Eclipse TE2000-U) and analyzed using Neurolucida software (MBF Bioscience). An average of 10 astrocytes from each animal with clear cell bodies and processes in both gray and white matter were chosen for reconstruction. The cells chosen were fully intact and did not have processes that touched the edges of the field. The resulting files generated by 2D reconstruction were analyzed with Neurolucida Explorer (MBF Bioscience), generating data of morphological measurements including cell area, branching points (nodes), arbor length, and volume (Figure 1A).

### Sholl analyses

Sholl analysis was performed on the data by placing concentric rings 10 μm apart around the cell starting from the center of the cell body and radiating outward. Intersections were determined as points where the astrocytic processes crossed a concentric ring. Branching points (nodes) are expressed as a quantity per concentric ring area.

### Statistical analyses

Statistical analyses were performed using GraphPad Prism (version 5, GraphPad Software, La Jolla, CA). Normality was assessed by Kolmogorov-Smirnov test, and data that passed normality were analyzed by Student's *t*-test. Data that were not distributed normally were assessed by Mann-Whitney test to determine significance between groups. Results are expressed as mean ± SEM. For all analyses, significance was set at *p* < 0.05.

## Results

### Infection with *B. melitensis*

Macaques were infected with *B. melitensis* bacteria by inhalation at a dose of between 6.88 × 10^5^−1.70 × 10^6^ cfu/animal. We postulated that *Brucella* could be isolated from multiple organs following aerosol infection. To address this question, we examined blood draws and biopsies at 1, 15 and 45 days postinfection. The data from this study are presented in Table 1. One day after infection, there were, on average 1,165 bacteria/ml in blood, and undetectable levels in bronchoalevolar lavage (BAL) fluid (Table 1). At the time of necropsy, 45 days postinfection, bacteria could not be cultured from blood, as anticipated (Nalini et al., 2012), but were cultured from BAL. Bacteria were also found in liver, spleen and bronchial lymph node.

### Summary of pathologic findings

The classic lesions of brucellosis, characterized by accumulation of epithelioid-type macrophages, small numbers of syncytial giant cells and lymphocytes with foci of necrosis and suppuration were seen in the inguinal node of one animal (II31). All animals had hyperplastic lymph nodes (6/6), and many had more subtle but less specific perivascular accumulations of lymphocytes and histiocytes in the lung and liver (5/6), salivary gland (3/6), brain and meninges, kidney, stomach (2/6), prostate, and urinary bladder (1/6). Lymphoid hyperplasia was noted in the spleen, small and large intestines (3/6).

It should be noted that the animal with a classic granulomatous lesion (II31) did not present with meningoencephalitis, and the two animals with meningoencephalitis (IG65, IP73) did not have significant granulomatous inflammation. However, all three and nearly all the rest of the cohort had mild inflammation in the lung and liver with generalized lymphoid hyperplasia.

### Induction of neurobrucellosis

Neurobrucellosis has been identified in approximately one-third of individuals with confirmed *Brucella* infection (Guven et al., 2013). Based on infections in animals and humans, we postulated that there would be a non-suppurative encephalitis present in brains of macaques infected with *B. melitensis*. To address this question, we examined 6 μm thick paraffin-embedded tissues stained with hematoxylin and eosin. The data from this study are presented in Figure 1. Two of the six animals infected with *B. melitensis* via aerosol had histologic evidence of encephalitis. Occasional lesions were evident in brains of these two macaques at 45 days postinfection (Figures 1A,B). These lesions were non-supperative in nature, and consistent with mild to moderate perivascular lymphocytic infiltration. The remaining four animals had no evidence of encephalitis in the sections examined (Figure 1C). Thus, the proportion of macaques with neurobrucellosis mirrors that recently described in humans infected with *Brucella*.

### *Brucella* infection upregulates TLR2 expression on astrocytes

**Figure 2:**
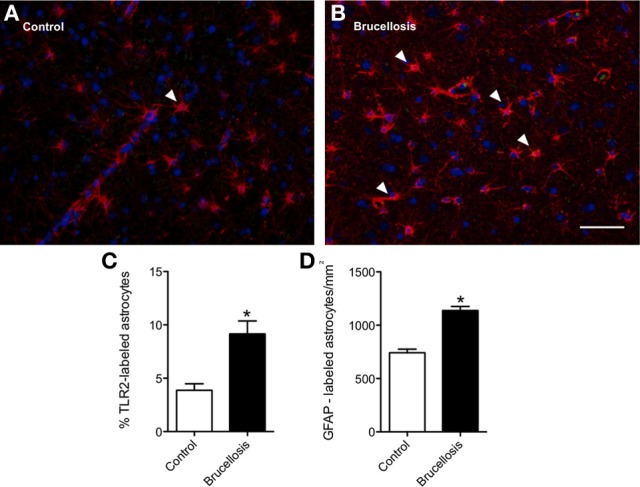
**Innate immune activation is coincident with increased astrocyte numbers in white matter**. In control macaques, very few GFAP-positive (red) astrocytes express TLR2 **(A**, green). Arrowheads demonstrate double positive cells. In contrast, more astrocytes express TLR2 following infection with *B. melitensis* **(B)**. To confirm if this increase was significant, the proportion of double labeled cells was calculated. 3.9% of white matter astrocytes were TLR2 positive **(C)**. This increased to 9.2% (*P* = 0.0008) with infection. This was coincident with an increased number of astrocytes within the white matter **(D)**. Due to the low expression of GFAP positive cells in cortical gray matter, the percentage of TLR2 and GFAP double-labeled astrocytes could not be evaluated quantitatively. Significance set at ^*^p < 0.05, Scale bar = 25 um.

The expression of TLR2 has been shown recently to be linked to the clearance of *Brucella* in lung, but not in the spleen nor the liver (Pei et al., 2012). We hypothesized that *Brucella* infection would induce increased Toll-like receptor (TLR) expression and other markers of activation on astrocytes. In order to test this hypothesis, we stained 6 μm paraffin sections with TLR2 and GFAP antibodies. Our approach was to count the number of GFAP positive cells (astrocytes) per mm^2^. We then determined what proportion of these cells were double positive for TLR2. White matter astrocytes in control macaques are rarely TLR2 positive (3.4%, Figure 2A). Forty-five days after macaques were infected with *Brucella*, this was increased to 7.7% in the TLR2 expression on astrocytes in white matter, (Figures 2B,C, *p* = 0.0008). The alterations in astrocyte activation could have been caused by either dividing or migrated astrocytes. To determine if there were altered numbers of astrocytes in frontal lobe of macaques infected with *Brucella*, we counted the number of GFAP positive cells per unit area. Macaques infected with *Brucella*, had increased numbers of white matter astrocytes per unit area compared with controls (1139/mm^2^ vs. 742/mm^2^ in controls, *p* < 0.0001, Figure 2D). Due to the low expression of GFAP positive cells in cortical gray matter, the percentage of TLR2 and GFAP double-labeled astrocytes could not be evaluated quantitatively. We have concluded from this experiment that there are increased numbers of astrocytes in white matter of macaques infected with *B. melitensis* and that these astrocytes also have increased innate immune activation.

### Astrocytic processes are extended following *Brucella* infection

**Figure 3:**
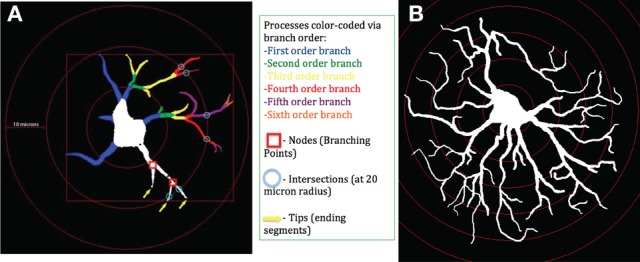
**Schematic of astrocytes and parameters measured**. Astrocytes were identified using GFAP antibody. Images were imported into Neurolucida and examined in Neurolucida explorer. At left, an astrocyte from a control macaque **(A)**. At right, an astrocyte from a macaque infected with *B. melitensis* **(B)**. Each concentric ring represents 10 μm.

Fluorescent images of astrocytes were imported into Neurolucida and analyzed. Representative tracings of astrocytes from control (Figure 3A) and infected **(B)** macaques demonstrate numerous changes. Each concentric ring represents 10 μm.

### Morphometric analyses of astrocytes reveals cell body hypertrophy and longer, more complex processes

**Figure 4:**
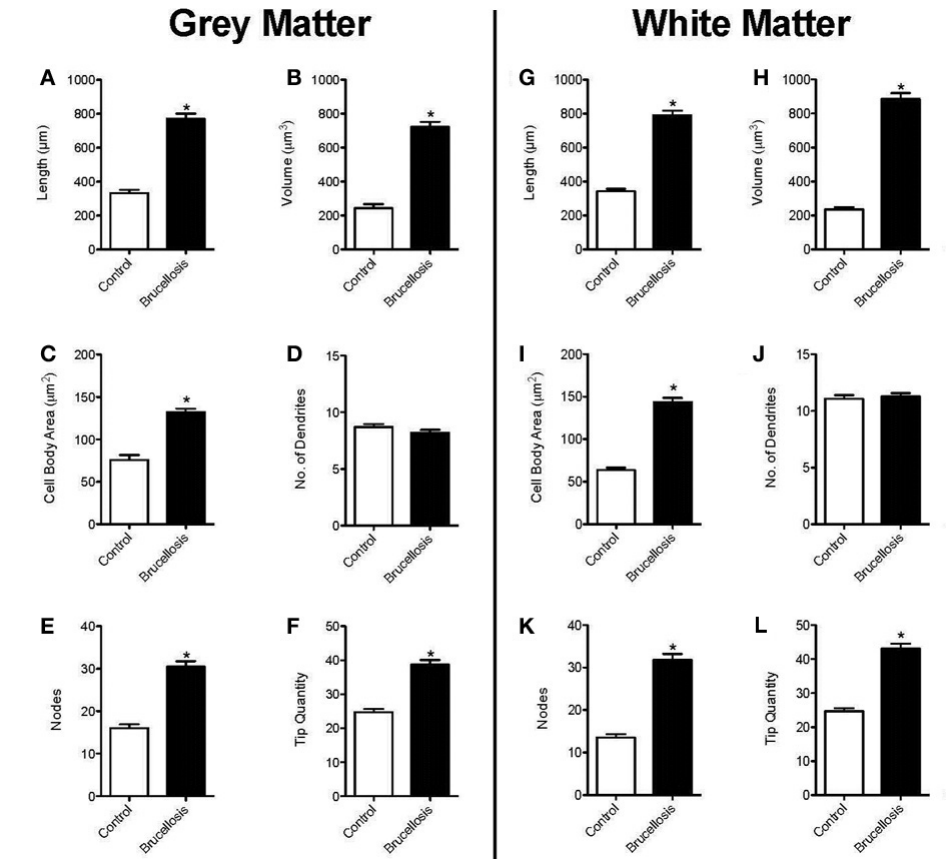
***B. melitensis* infection induces astrocyte hypertrophy**. The total length of processes of astrocytes from infected macaques were increased in both gray and white matter **(A, G)**. Similarly, the volume of the processes was increased **(B, H)**. This was coincident with a hypertrophied cell body **(C, I)**. It was curious that there were no changes in the number of primary processes **(D, J)**. Therefore, the increased arbor was a result of increased branching **(E, K)** and thus, tip quantity **(F, L)** in astrocytes of macaques infected with *Brucella*. ^*^p < 0.05.

To determine how this activation of the astrocytes was reflected in the overall morphology, we imported fluorescent images of GFAP positive cells into Neurolucida. We then analyzed the morphology of the cells to assess total length and volume of processes combined with cell body area as a measure of hypertrophy. The complexity of the astrocytes was also assessed by counting the number of dendrites, nodes and tips per astrocyte. These data are presented in Figure 4. The sum of astrocyte process lengths radiating from cell bodies to the end of processes was calculated for each astrocyte in gray (Figure 4A) and white matter (Figure 4G). The cell arbor was increased greater than two-fold in animals infected with *B. melitensis*. This change was measured in both gray (*Brucella*: 769.9 ± 30.97 μm^2^ vs. Control: 332.1 ± 20.08 μm^2^, *p* < 0.0001) and white matter astrocytes (*Brucella*: 790.2 ± 28.01 μm^2^ vs. Control: 342.9 ± 14.32 μm^2^, *p* < 0.0001).

Using the diameter of the astrocyte processes, frusta were created in Neurolucida to determine the arbor volume of the astrocyte processes. The arbor volume of the astrocyte processes was also increased significantly in gray or white matter astrocytes of animals infected with *Brucella* compared with control brains (Figure 4B,h: *p* < 0.0001 in both cases). Thus, as the cell arbor was significantly increased, in addition to the volume, this shows that processes of astrocytes in *Brucella* animals are not thickened. These changes in astrocyte morphology could be expected to affect the volume and composition of the extracellular space and, as a result, neuronal activity (Shao et al., 1994).

### *B. melitensis* induces cell body hypertrophy

To assess cell body atrophy or hypertrophy, we measured the area of the cell body of astrocytes, as determined in Figure 3. The cell bodies in control gray matter astrocytes were 75.75 ± 5.765 μm^2^ (Figure 4C). There was a significant increase in cell body area in gray matter astrocytes of *Brucella* infected macaques (132.3 ± 4.04 μm^2^), with a larger increase in white matter (63.77 ± 2.89 μm^2^ vs. 143.7 ± 4.89 μm^2^, Figure 4I), indicating cytoplasmic expansion/cellular swelling.

### *B. melitensis* does not induce *de novo* process formation

We quantified the number of processes leaving the cell body (shown as number of dendrites, Figures 4D,J). There was no significant change in the number of dendrites in either gray (*p* = 0.108) or white matter (*p* = 0.458) following infection with *Brucella*. The number of bifurcations (nodes) increased significantly between control and *Brucella* gray (*p* < 0.0001) and white matter (*p* < 0.0001) astrocytes (Figures 4E,K, respectively). Additionally, there was increased numbers of process end points (tip quantity) in *Brucella* gray (Figure 4F, *p* < 0.0001) and white matter astrocytes (Figure 4L, *p* < 0.0001) compared with controls. Therefore, the complexity of astrocytes was increased dramatically in the brains of macaques infected with *B. melitensis*.

### Sholl analyses

**Figure 5:**
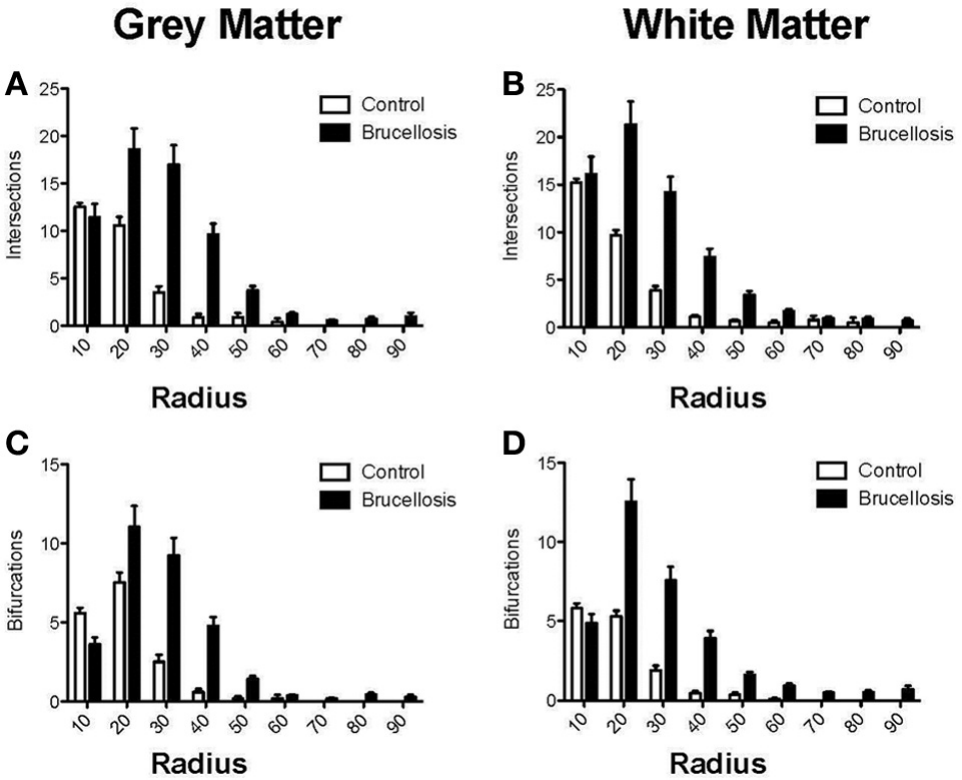
**Astrocytes have increased complexity following *Brucella* infection**. In both gray and white matter astrocytes there was an increase in the number of intersections from 20 μm onwards (**A, B**, respectively). This was borne out by the increased number of bifurcations in the astrocyte processes **(C, D)** leading to increased complexity of the astrocytes.

Based on previous studies (Lee et al., 2013), we hypothesized that the majority of these observed changes would occur closer to the cell body than distally. To answer this question, we performed a modified Sholl analysis where concentric 10 μm diameter circles were superimposed on the Neurolucida traces. Our approach was then to quantify the number of intersections and bifurcations per 10 μm increase in radius. There was no significant change in the number of intersections or bifurcations within the first 10 μm radii around the cell body of *Brucella* gray or white matter astrocytes (Figures 5A–D), perhaps due to cell body hypertrophy. However, there were significant increases in each of these parameters between 20 and 50 μm. It was noted that there were very few if any intersections or bifurcations in control astrocytes beyond 50 μm from the cell bodies, again showing that the processes were very much longer and more complex in astrocytes of animals infected with *Brucella*.

Overall, we observed increased numbers of astrocytes per unit area of white matter in astrocytes of animals infected with *B. melitensis*. These astrocytes had increased TLR2 expression, suggestive of innate immune activation. This was combined with a dramatic increase in process length (cell arbor and radius) in gray and white matter astrocytes in primates infected with *Brucella*, (schematically represented in Figure 3). Sholl analysis showed increased complexity of *Brucella* gray and white matter astrocytes. Furthermore, astrocytes in gray and white matter from animals with *Brucella* showed an increase in tip quantity: a further indication of increased astrocyte complexity following systemic infection with *Brucella*.

In summary, these data confirm that astrocytes are activated in multiple manners in the context of *Brucella* infection. There are alterations in morphology, intermediate filament expression and innate immune activation. It was interesting that none of these changes was specific to gray or white matter, however.

## Discussion

Brucellosis is an occupational zoonosis in shepherds, veterinarians, dairy industry professionals and abattoir workers in much of the world (Boschiroli et al., 2001; Pappas et al., 2005) and endemic in sub-Saharan Africa and the Middle East and South America. Because of it's high morbidity, and low infectious dose, combined with low technical requirements involved with culturing, *Brucella* is considered an agent that holds the potential for misuse, and thus possession and research activities are highly regulated by the US CDC and USDA. (Pappas, 2010; Pei et al., 2012). Approximately one-third of individuals infected with *Brucella* display neurological complications (Guven et al., 2013) including behavioral changes, psychosis, dementia, meningitis and physical changes apparent by CT and MRI (Nalini et al., 2012; Guven et al., 2013; Montazeri et al., 2013; Rajan et al., 2013).

Neurobrucellosis has been observed in approximately one-third of individuals infected with *Brucella* (Guven et al., 2013). In the present study, 2 of 6 animals had histologic evidence of encephalitis 6 weeks after initial aerosol infection (Figure 1), mimicking what is been clinically observed. We were interested in determining the underlying mechanisms of neuropathology. Our recent studies in other diseases have shown specific patterns of astrocyte activation combined with increased TLR2 protein expression [manuscripts under review, revision and Lee et al. (2013)]. Thus, we set out to quantify astrocyte activation with regard to innate immune activation, proliferation/migration and morphology.

White matter astrocytes in macaques infected with *B. melitensis* demonstrated increased expression of TLR2 compared with controls, suggesting increased innate immune activation. The role of TLRs 2 and 4 in the clearance of aerosolized *Brucella* in the mouse model has been partially elucidated and has demonstrated that TLR2 plays a role in the clearance of the bacteria from the lung but not from the spleen and liver (Pei et al., 2012). It is therefore possible that the increased TLR2 expression may have a role in controlling *Brucella* infection in brain, although that is beyond the scope of this preliminary study. Conversely, stimulation of TLR2 on astrocytes has been shown recently to induce neurotoxicity through release of reactive oxygen intermediaries (Ma et al., 2013). In white matter there were also increased numbers of astrocytes indicative of either proliferation or migration from other areas. That there were considerably more GFAP-immunopositive astrocytes in white matter than in control animals suggest either cell migration out of the gray matter, or possibly astrocyte proliferation. Combined, these data confirm a role for astrocytes in the innate immunity within brain (Crews et al., 2011).

Astrocytes display dynamic plasticity in their distal processes in response to changes in their extracellular environment (Safavi-Abbasi et al., 2001). Until now, astrocytosis has been considered a non-specific reaction to infectious and non-infectious processes in the brain. It is possible that the astrogliosis observed may be related to the perivascular cuffing and/or the meningitis observed in the animals infected with *B. melitensis* (Figure 1). We have previously shown that rhesus macaques infected with simian immunodeficiency virus have activated astrocytes with increased TLR2 expression and altered morphology, even in the absence of active lesions in brain (Lee et al., under revision). However, in these other studies, the astrocytes displayed considerable shorter and thicker processes, combined with much lower complexity. Indeed, the morphologies of the astrocytes in macaque infected with *Brucella* were more similar to those observed in depressed suicidal humans (Torres-Platas et al., 2011).

The extreme hypertrophy observed, not only in the cell body but also of all morphologic parameters tested (with the exception of the number of primary processes) is beyond that observed with either aberrant behavior (Torres-Platas et al., 2011) or lentiviral or flaviviral infections (Lee et al., under revision). We have recently shown that morphological changes in astrocytes have direct changes in other physiological responses, including secretion of proinflammatory cytokines and expression of integrins (Renner et al., 2013). It is curious that, in white matter, there was an increase in both number and size of GFAP-immunopositive astrocytes observed. Increased numbers of larger astrocytes would decrease the volume and alter the composition of the extracellular space between astrocytes and, as a result, neuronal activity (Shao et al., 1994). Thus, it is becoming apparent that the morphology of astrocytes may be determined by the pathologic agent involved, be it bacterial (*Brucella*), lentiviral (SIV), flaviviral (Dengue) or even noninfectious causes [behavioral (Lee et al., 2013)].

There could also be anticipated to be effects on synaptic integrity and the blood-brain barrier (BBB) during neurobrucellosis. Recent studies have demonstrated that synaptic stability is linked to expression of TLR2 within brain (Freria et al., 2012). Our data showed that increased TLR2 expression is correlated with infection with *Brucella*, indicating a possible loss of synaptic stability in white matter. Loss of astrocyte processes leads directly to loss of BBB integrity (Willis et al., 2004a,b). As we observed dramatically increased branching and tips in the astrocytes, the opposite may be happening: the early responses by astrocytes could be a reaction to the presence of bacteria, and may reflect an attempt to close the cerebral microvasculature against penetration of bacteria into the parenchyma (Zlotnik, 1968). It would therefore be very interesting to examine the astrocyte responses to other bacterial infections of brain to determine if these responses observed are unique to *Brucella* infection, or are a more general response to bacteria attempting to enter the brain.

At the time of necropsy (45 days postinfection) there were undetectable levels of bacteria in blood. However, bacteria were detected in BAL fluids (Table 1) and other organs including spleen, liver and lymph nodes, in agreement with previous studies in humans (Guven et al., 2013) and rhesus macaques (Henning et al., 2012). Thus, although bacteria were not found in circulation 45 days after infection, there was evidence of bacteria within a number of organ systems in the infected macaques. The lack of culture of *Brucella* in CSF cannot be taken as diagnostic for the absence of neurobrucellosis, as less than 20% of cases have positive cultures (Nalini et al., 2012; Guven et al., 2013).

It will be interesting to determine if therapeutics or putative vaccine candidates prevent this astrocyte activation, or if the morphology would return to that observed in control animals following treatment as was observed recently with neuronal morphology (Stagni et al., 2013). It is also then possible that the observed astrocyte activation is an acute response to bacterial infection that would revert to a more normal phenotype as the infection is cleared by the adaptive immune response.

## Author contributions

All authors contributed to the writing of the manuscript. Peter J. Didier performed pathologic analyses, Kim M. Lee, Hope A. Sansing, Kevin B. Chiu performed immunohistochemistry, fluorescence and Neurolucida studies, under the guidance and supervision of Andrew G. MacLean. Thomas A. Ficht, Angela M. Arenas-Gamboa, and Chad J. Roy designed the infection studies.

## Conflict of interest statement

The authors declare that the research was conducted in the absence of any commercial or financial relationships that could be construed as a potential conflict of interest.
